# CDKL3 Targets ATG5 to Promote Carcinogenesis of Esophageal Squamous Cell Carcinoma

**DOI:** 10.3389/fonc.2020.01602

**Published:** 2020-08-21

**Authors:** Suna Zhou, Mingxin Zhang, Chao Zhou, Wei Wang, Haihua Yang, Wenguang Ye

**Affiliations:** ^1^Laboratory of Cellular and Molecular Radiation Oncology, The Affiliated Taizhou Hospital, Wenzhou Medical University, Taizhou, China; ^2^Department of Radiation Oncology, The Affiliated Taizhou Hospital, Wenzhou Medical University, Taizhou, China; ^3^Department of Gastroenterology, The First Affiliated Hospital of Xi’an Medical University, Xi’an, China; ^4^Department of Gastroenterology, The Affiliated Taizhou Hospital, Wenzhou Medical University, Taizhou, China

**Keywords:** cyclin-dependent kinase-like 3, autophagy-related gene 5, esophageal squamous cell carcinoma, prognosis, carcinogenesis

## Abstract

**Objective:**

Our previous study suggested cyclin-dependent kinase-like 3 (CDKL3) acts as a new oncogene in esophageal squamous cell carcinoma (ESCC) cell line TE-1. However, the molecular mechanisms and biological effects of CDKL3 in ESCC remain unknown. In the present study, we aimed to explore the clinical significance of CDKL3 in ESCC and how CDKL3 regulates the malignant behavior of ESCC.

**Methods:**

ESCC samples were stained by immunohistochemical staining (IHC) and analyzed for the expression of CDKL3. The functions of CDKL3 on proliferation, apoptosis, migration, invasion, and colony formation were investigated by celigo assay, MTT assay, colony formation, caspase 3/7 activity analysis, transwell migration and invasion assay, respectively. A transplanted tumor model was established to study the functions of CDKL3 on the tumorigenesis of ESCC cells. Microarray analysis was utilized to identify the CDKL3-regulated genes in ESCC cells.

**Results:**

ESCC tumor tissues possessed a significantly higher expression of CDKL3 and autophagy-related gene 5 (ATG5) than matched adjacent normal tissues. The high expressions of CDKL3 were positively associated with the tumor-node-metastasis (TNM) stage and Ki67. Upregulated ATG5 expression was positively correlated with male, advanced tumor (T) stage, and TNM stage. Kaplan-Meier analysis showed that ESCC patients with higher expression of CDKL3 or ATG5 had a shorter overall survival. The worst prognosis was recognized in patients with both high manifestations of CDKL3 and ATG5. Time-dependent receiver operating characteristic (ROC) curve was established to reveal that the combination of CDKL3, ATG5, and TNM stage–based model had better prognostic accuracy than TNM stage. Moreover, CDKL3 knockdown markedly repressed cell growth and aggressivity *in vitro* and *in vivo*. Mechanistically, ATG5 was confirmed as a downstream gene involved in the pro-oncogenic function of CDKL3.

**Conclusion:**

CDKL3 can be utilized as an independent poor prognostic marker in ESCC patients. Furthermore, CDKL3 may promote tumor profession, invasion, metastasis, and prohibit tumor apoptosis partly by ATG5 activation.

## Introduction

Esophageal cancer is the seventh most lethal cancer in the world (1). Esophageal squamous cell carcinoma (ESCC) is the primary pathological type of esophageal cancer in Asia, accounting for more than 80% of patients with esophageal cancer in China, and the 5-year survival rate is about 20.9% (2). ESCC occurrence and progression is a sophisticated program regulated by diverse genes and signaling. Therefore, identifying and studying novels oncogenes related to the progress of ESCC will help to develop new prognostic indicators and therapeutic targets. In our previous study, we find that cyclin-dependent kinase-like 3 (CDKL3) acts as a new oncogene in ESCC cell line TE-1 and overexpression of CDKL3 predicts shorter overall survival in ESCC patients from the TCGA dataset (3). However, further researches are required to uncover the specific role and precise mechanism of the regulation of CDKL3 in the malignant transformation of ESCC.

Autophagy is a cellular self-protection process by which it escapes from external stress via metabolizing intracellular proteins and organelles and serves an essential role in carcinogenesis and progression (4, 5). ATG5 is identified as a critical autophagy gene, contributing to the malignant feature of cancer cells, and its higher expression predicts poor prognosis in patients with early-stage ESCC (6–8). However, the association between ATG5 and CDKL3 in cancer has not been studied previously. Therefore, a series of studies were designed to illustrate the role and regulatory mechanism of CDKL3 in ESCC progression. We examined the association between the expression of CDKL3, ATG5, and clinicopathological features, as well as over survival. Besides, the role of CDKL3 in ESCC was studied *in vitro* and *in vivo* assays, and to exploit whether ATG5 exerts its effects on CDKL3-dependent cancer promotion.

## Materials and Methods

### Tissues and Immunohistochemical Analysis

Samples were taken from patients with ESCC (*n* = 103) who underwent surgery at The Affiliated Taizhou Hospital of Wenzhou Medical University (Zhejiang, China) from January 2006 to December 2008. The samples contained 103 ESCC tumor tissues and 77 normal esophageal tissues. The Institutional Review Board of Taizhou Hospital approved the study and informed consent was obtained from each patient. Immunohistochemical staining (IHC) was performed as follows. Briefly, tissue sections were cut, deparaffinized and rehydrated, and incubated with the rabbit polyclonal anti-CDKL3 antibody and rabbit polyclonal anti-ATG5 antibody (Proteintech, Chicago, IL, United States) at 1/1000 and 1/750 dilutions, respectively. The expression of CDKL3 and ATG5 was scored by the percentage and intensity of positively stained cells. In this study, patients were assigned to the low expression group (final staining score ≤4) or the high expression group (score >4). For Ki67, P53, and PDL-1, tumors in which the positively stained area covered >5% were classified as positive cases. The intensity of gene expression was assessed by two independent observers who were blinded to the objective of this study.

### Cell Lines

Human ESCC cell lines, TE-1 and KYSE-150 purchased from Shanghai Cell Biology Institute of Chinese Academy of Sciences (Shanghai, China) were cultured in RPMI 1640 supplemented with 10% FBS (Gibco, Grand Island, NY, United States) at 37°C with 5% CO2.

### Cell Transfection

TE-1 and KYSE-150 cells at the logarithmic growth phase were transfected with the recombinant lentiviral vector (LV) named as KD (LV expressing CDKL3-shRNA) labeled with green fluorescent or NC-1 (LV expressing scrambled shRNA) labeled with green fluorescent or OE (LV expressing ATG5-mimic) labeled with red fluorescent or NC-2 (LV expressing scrambled mimic) labeled with red fluorescent according to the manufacturer’s instructions (GeneChem Corporation, Shanghai, China). Then, the infection efficiency was detected by a fluorescence microscope (Olympus Corporation, Tokyo, Japan) at 72 h after infection.

### Western Blot Assay

Briefly, the total protein in ESCC cells was extracted, followed by protein quantification using BCA Protein Assay Kit (Beyotime, Shanghai, China). 10% SDS-PAGE was used to separate protein samples. After transferring onto PVDF membranes (Millipore, Burlington, MA, United States), followed by blocking with 5% skim milk for 1 h and then separately incubated with mouse anti-CDKL3 antibody (Sigma-Aldrich, St. Louis, MO, United States) or mouse anti-ATG5 antibody (Santacruz, Santa Cruz, CA, United States) or rabbit anti-LC3A/B antibody (Cell Signaling Technology, Danvers, MA, United States) at dilutions of 1:1000 or 1:500 or 1:1000 overnight. Mouse anti-GAPDH (Santacruz, Santa Cruz, CA, United States) antibody (1:2000) was used as an internal control at 4°C overnight. Lastly, the visualization of protein bands was realized using a horseradish-peroxidase (HRP)-conjugated IgG secondary antibody (Santacruz, Santa Cruz, CA, United States).

### Cell Proliferation Assay

After infection, ESCC cells were seeded into a 96-well plate at 2000 cells per well. The Celigo imaging cytometer (Nexcelom Bioscience LLC, Lawrence, MA, United States) was used to capture live cells daily for 5 days. On the other hand, cell viability was detected at 24 to 120 h by MTT assay at an absorbance of 490 nm.

### Cell Apoptosis Assay

Esophageal squamous cell carcinoma cells, at a density of 20000 cells/well, were seeded into the six-well plate after infection. All cells at 70% confluency were harvested and stained with Annexin-V-APC (eBioscience, San Diego, United States). Then, an inverted fluorescence microscope (IX71, Olympus, Japan) was applied to detect apoptosis cells. On the other hand, ESCC cells were seeded into a 96-well plate at a density of 20000 cells/well followed by 24 h incubation. Then, the Caspase-Glo 3/7 Assay (Promega, Madison, WI, United States) was used for apoptosis detection following the manufacturer’s instructions.

### Cell Migration and Invasion Assays

After infection, ESCC cells at a density of 80000 cells/well were seeded on a polycarbonate membrane either coated with or without Matrigel insert in a Corning^®^ BioCoat^TM^ Matrigel^®^ Invasion Chambers System (Corning Costar, Cambridge, MA, United States). RPMI-1640 medium containing 30% FBS was added to the lower chamber. After 17–40 h incubation, wiped the cells on the top surface of the upper chamber by a cotton swab. The invasive/migrated cells were stained with crystal violet in aqueous solution for 5 min, followed by cell counting under a microscope and photograph.

### Cell Colony Formation Assay

At a density of 1000 cells/well, transfected cells were seeded into the six-well plate. After 8–14 days incubation, cells were fixed with 4% paraformaldehyde (Sigma-Aldrich, Springfield, MO, United States) for 30 min and stained with crystal violet. Afterward, colonies were counted under light microscopy and photographed under a fluorescence microscope.

### Tumor Xenograft Formation Assay

A xenograft tumor was established to study the functions of CDKL3 *in vivo*. Briefly KYSE-150-NC cells (KYSE-150 cells stably expressing scrambled shRNA) and KYSE-150-KD cells (KYSE-150 cells stably expressing CDKL3-shRNA) (4 × 10^6^ cells) were transplanted subcutaneously in the right-back flank of 4-week-old male BALB/c nude mice (Shanghai SLAC Laboratory Animal Co., Ltd., Shanghai, China). After transplantation, tumor size [(π/6) × (length) × (width)^2^] and weight were measured every other 3 days for 2 weeks until the mice were humanely sacrificed.

### Microarray Analysis

We analyzed the gene expression profiles of three pairs of KYSE-150-shCtrl and KYSE-150-shCDKL3 cells by GeneChip^®^ PrimeView^TM^ Human Gene Expression Array. Firstly, a signal histogram, relative signal box plot, correlation analysis, and principal component analysis were performed to assess the quality of the microarray data. Supplementary Figure S1A signal histogram showed that the signal intensity with the average Z-score of all samples in this project is less than 2, suggesting the reliability of all the chip data. Supplementary Figure S1B relative signal box plot showed that the median Z-score of all samples in this project is less than 2, which indicates that the excellent repeatability of chip experiments. Supplementary Figure S1C correlation analysis described the minimal differences in gene expression among the respective two groups and the max differences between the shCtrl and shCDKL3 group. Supplementary Figure S1D principal component analysis displayed the minimal differences among the respective two groups and the max differences between the two groups. In this project, we used a linear model based on the Bayesian model to calculate the *P*-value and the Benjamini–Hochberg method was performed to correct the significant difference level (FDR).

### Quantitative Real-Time Polymerase Chain Reaction (qRT-PCR)

To confirm the up-regulated and down-regulated genes selected from microarray analysis, total RNA was isolated using Trizol (Invitrogen, Carlsbad, CA, United States). After the cDNA synthetization, qRT-PCR was performed on a LightCycler 480 II RT-PCR System (Roche, Basel, Switzerland) with SYBR Master Mixture (TAKAR, Kyoto, Japan). PCR conditions were as follows: 95°C for 40 s, and then 40 cycles of 95°C for 5 s and 61°C for 40 s. The relative gene expression was measured using the ΔΔCt method. GAPDH was used as an internal reference. The primers used in study were as follows: CCL5: forward 5′-AGCCCTCGCTGTCATCCTCAT-3′, reverse 5′-CCTGCCAGACTTGCTGTCCCT-3′; SAMD8: forward 5′- TGGCTCCTGGTTCTTCTTCTT-3′, reverse 5′- CAGTCAGG GTCATACCAAAGC-3′; PLAUR: forward 5′- CCTGAGCTATC GGACT GGC-3′, reverse 5′- AGGTAACGGCTTCGGGAAT-3′; SERPINE2: forward 5′- AACGCCGTGT TTGTTAAGAATG-3′, reverse 5′-CGTGATTTCCACAGACCCTTG-3′; BIK: forward 5′-GGAGG ACTTCGATTCTTTGG -3′, reverse 5′-GAAACCG TCCATGAAACTTCTA-3′; DRAM2: forward 5′- GCAGCAG AATGGTCTATGTCA-3′, reverse 5′- AGCCGTGTTCGTTCAT TGTTA-3′; RNF41: forward 5′- GCTGTCCTG CTTCTATT GTCA-3′, reverse 5′- TAGCGTCGGTTCATCTGTCTA-3′; UT P18: forward 5′- CTCCAAGAAACAAACCCAAAG-3′, reverse 5′-ATGGTGCAACCTATACA TCAGG-3′; STK38: forward 5′- AGCAACCTTATCGCTCAACAT-3′, reverse 5′- TTTCTT CTGA ACAAGCCGTAC-3′; SMAD5: forward 5′- GCCAA AGAATCCCAAGT-3′, reverse 5′- GGGCAG GTAGATACA AACA-3′; WNT10A: forward 5′- ATCGCCTTGGCTCTTGGG-3′, reverse 5′- GG AGACAGACTGACGGGTTGC-3′; CCNG1: forward 5′- GCAACTGACTTGATCCGAATA-3′, reverse 5′- CA CACCTTCTCCAATACAATCT-3′; SLC35A5: forward 5′- TTTC AGTCACATCCG TCTT-3′, reverse 5′- TGGTTCCCTTCC TT CAGTA-3′; ATG5: forward 5′-AAGAGTGTTTATTC GTC GGT-3′, reverse 5′-ATCA CAGCTTAGTGTTCCCT-3′; LMNB1: forward 5′-AAGCATGAA ACGCGCTTGG-3′, reverse 5′- AGT TTGGCATGGTAAGTCTGC-3′; PARP1: forward 5′-TCTG AGCTTCGGTGGGATGA-3′, reverse 5′-TTGGCATACTCTG CTGCAAAG-3′; CDK6: forward 5′- TCTTCATTCACACCG AGTAGTGC-3′, reverse 5′-TGAGGTTAGAGCCATCTGGAAA-3′; BCL2L11: forward 5′-TAAGTTCTGAGTGTGACCGAGA-3′, reverse 5′- GCTCTGTCTGTAGG GAGGTAGG-3′.

### Statistical Analysis

All investigations were repeated at least three times. The Student *t*-test and Mann–Whitney *U* test was used to analyze differences between the groups. The Kaplan–Meier method and the log-rank test were applied for survival analysis and comparison of survival curves. Cox proportional hazards regression was performed to analyze the prognosis of CDKL3 and ATG5 in univariate and multivariate models. In addition, the time-dependent ROC curve at 5y-OS and the area under the curve (AUC) value were implemented to assess the predictive accuracy of the CDKL3 and ATG5–based prognostic model in ESCC patients. The results are presented as the mean ± SD in this study. *P* < 0.05 was designated as a statistical significance value.

## Results

### CDKL3 Knockdown Inhibits Cellular Proliferation

**FIGURE 1:**
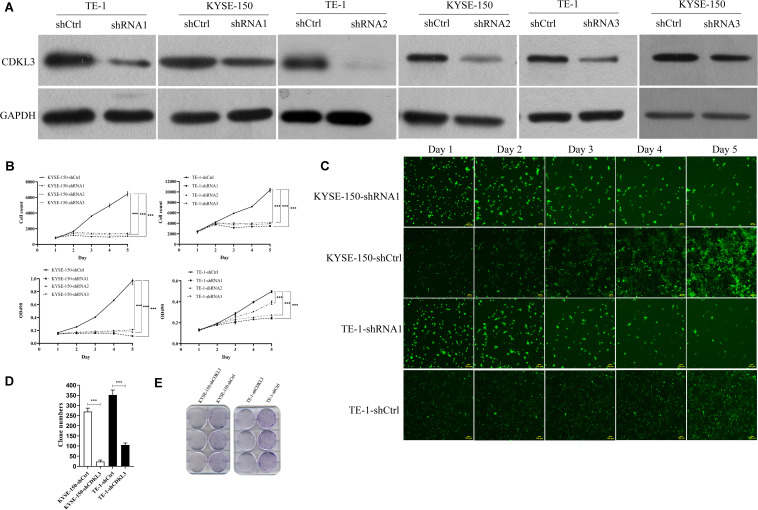
Knockdown of CDKL3 inhibited cellular proliferation and the colony formation ability in TE-1 and KYSE-150 cells. **(A)** Western blot analysis showed that LV-shCDKL3 significantly downregulated protein levels of CDKL3 compared with LV-shCtrl group after 72 h infection. **(B)** The Celigo assay and MTT assay confirmed the inhibitory effect of CDKL3 knockdown on the proliferation rate of TE-1 and KYSE-150. **(C)** The typical Celigo images for the effect of CDKL3-knockdown on the proliferation of TE-1 and KYSE-150 cells. **(D)** CDKL3 knockdown decreased the colon numbers in TE-1-shCDKL3 and KYSE-150-shCDKL3 cells compared to TE-1-shCtrl and KYSE-150-shCtrl cells, respectively. **(E)** colonies of ESCC cells stained with crystal violet. Data were represented as the mean ± SD of three separate experiments. ***P < 0.001.

We generated stable CDKL3 knockdown ESCC cells by using lentivirus-mediated shRNA1/2/3 and analyzed the effect on cellular proliferation. Western blot analysis confirmed stable knockdown of CDKL3 expression in TE-1-shRNA1/2/3 and KYSE-150-shRNA1/2/3 groups with a comparison to the control group, respectively (Figure 1A). The results of Celigo image cytometry and MTT assay confirmed that CDKL3 knockdown could effectively hinder the proliferation of TE-1 and KYSE-150 cells (Figures 1B,C). Comparing growth inhibitory effect among the three shRNA groups, the shRNA1 group exhibited a maximum inhibition efficiency (Figure 1B). Accordingly, shRNA1 group was selected to be used in further research. The effect of CDKL3 expression on the colony formation ability was detected via colony formation assay. As a result, the high reduction of clone counts was investigated in TE-1-shCDKL3 and KYSE-150-shCDKL3 cells (Figures 1D,E, *p* < 0.001), which verified the significant growth inhibition of CDKL3 knockdown in colony formation of ESCC cells.

### CDKL3 Knockdown Promotes Cellular Apoptosis

**FIGURE 2:**
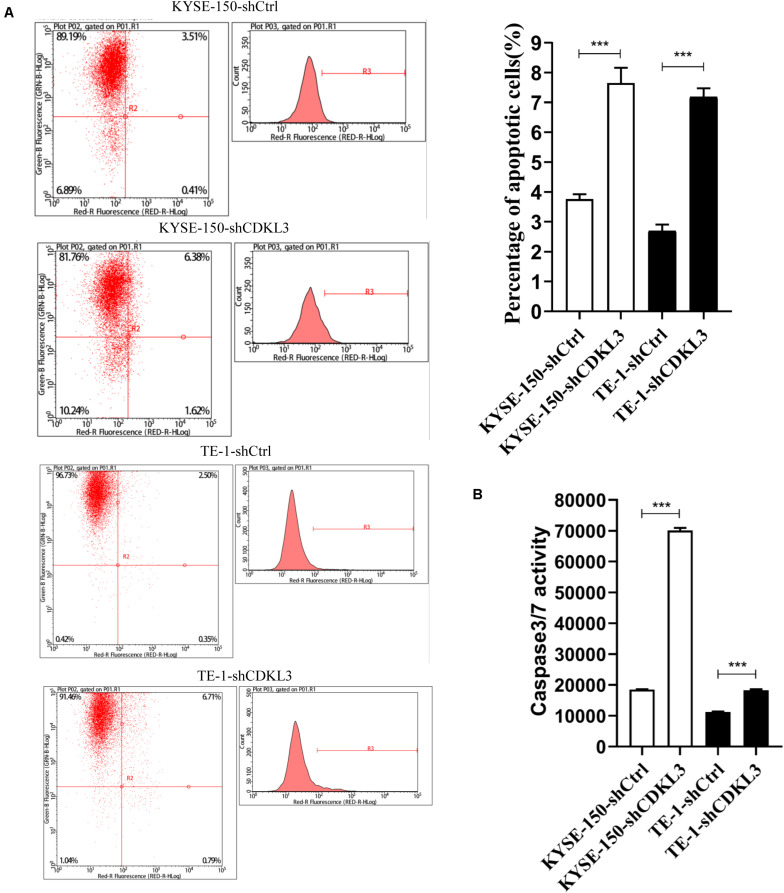
Knockdown of CDKL3 increased apoptosis in TE-1 and KYSE-150 cells, which was measured by Annexin-V-APC and caspase 3/7 assay. **(A)** Early apoptotic cells (Annexin-V+) were displayed in the upper and lower right quadrant. Knockdown of CDKL3 increased the early apoptotic rates in TE-1-shCDKL3 and KYSE-150-shCDKL3 cells. **(B)** Knockdown of CDKL3 remarkably enhanced caspase-3/7 activity in TE-1-shCDKL3 and KYSE-150-shCDKL3 cells. Data were represented as the mean ± SD of three separate experiments, ***P < 0.001.

The impact of CDKL3 on apoptosis of TE-1 and KYSE-150 was further investigated by flow cytometry and caspase-3/7 assay. Knockdown of CDKL3 resulted in an increase of apoptotic cell rate in TE-1-shCDKL3 and KYSE-150-shCDKL3 groups compared with their respective control groups (Figure 2A, *p* < 0.001). Furthermore, the caspase-3/7 assay showed that caspase-3/7 activity was significantly elevated in the CDKL3 knockdown groups as compared to the controls, indicating that apoptosis could be induced by CDKL3 inhibition (Figure 2B, *p* < 0.001).

### CDKL3 Knockdown Reduces Cellular Migration and Invasion

**FIGURE 3:**
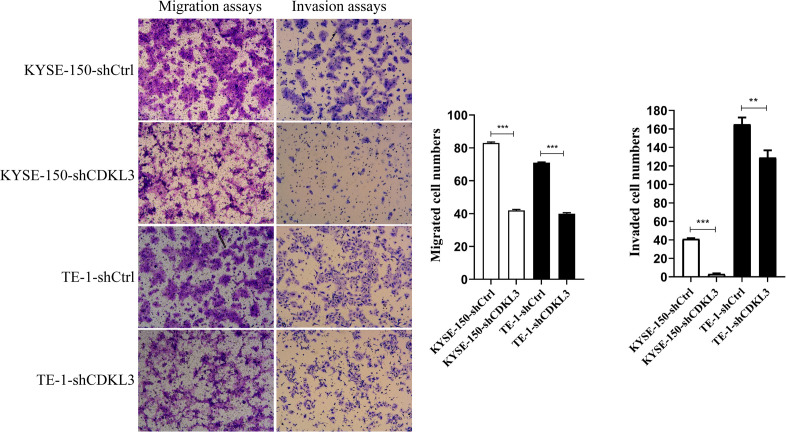
Using transwell-with or without Matrigel assay, CDKL3 knockdown decreased the numbers of migrated and invaded cells in the shCDKL3 group compared with the shCtrl group. Data were represented as the mean ± SD of three separate experiments. **P < 0.01, ***P < 0.001.

Cell migration and invasion assays were conducted to confirm whether CDKL3 is pivotal in ESCC cell metastasis. As shown in Figure 3, the total number of migrated and invaded cells was significantly reduced in the CDKL3 knockdown group with a comparison to the control group (*p* < 0.001), indicating that CDKL3 expression can regulate the capabilities of migration and invasion in ESCC cells.

### CDKL3 Knockdown Suppressed Tumorigenesis in Nude Mice

**FIGURE 4:**
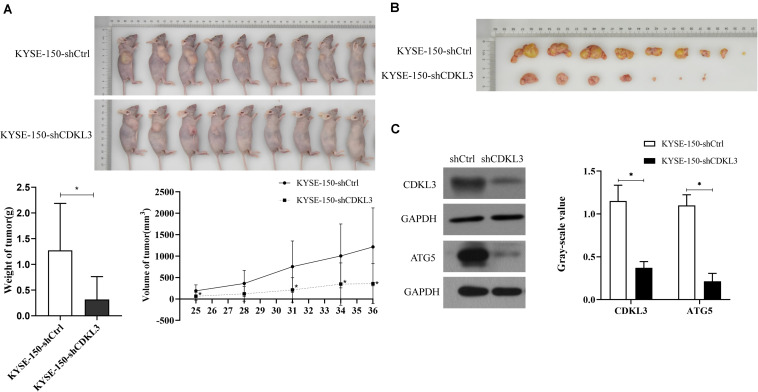
CDKL3 knockdown retarded tumor growth *in vivo*. **(A)** KYSE-150-shCDKL3 cells generated smaller tumors in nude mice compared to the KYSE-150-shCtrl cells, *P < 0.05. **(B)** KYSE-150-shCDKL3 cells formed lighter tumors compared to the KYSE-150-shCtrl cells, *P < 0.05. **(C)** Western blotting analysis of the presence of CDKL3 and ATG5 proteins from the harvested tumors as described for **(B)**, *P < 0.05. Data were represented as mean ± SD.

The tumorigenicity of KYSE-150-shCDKL3 and KYSE-150-shCtrl cells were further examined *in vivo*. CDKL3 knockdown and control cells were collected and inoculated subcutaneously into the right-back flank of the nude mice. Eventually, we discovered tumor formation from 7 out of 9 (77.8%) KYSE-150-shCDKL3 xenograft mice but 9 out of 9 (100%) control group. Furthermore, smaller volume and lighter weight of tumors were detected in KYSE-150-shCDKL3 xenograft mice than those in KYSE-150-shCtrl xenograft mice (Figures 4A,B, *p* < 0.001). In the tumors derived from KYSE-150-shCDKL3 cells, the protein expression of CDKL3 was successfully knocked down with ATG5 protein expression declining (Figure 4C). These results from Vivo assay suggested that the vital function of CDKL3 in the generation and progression of ESCC.

### Gene Expression Profile Analysis Revealed ATG5 Was a Downstream Target of CDKL3

To elucidate the molecular mechanisms through which CDKL3 promotes the proliferation of ESCC cancer cells, we analyzed the differential gene expression profiles between KYSE-150-shCtrl and KYSE-150-shCDKL3 cells by Gene Expression Array. As compared to the KYSE-150-shCtrl cells, 136 up-regulated genes and 192 down-regulated genes were identified in KYSE-150-shCDKL3 cells. A Scatter plot and Volcano plot was depicted based on the validated quality of microarray data.

**FIGURE 5:**
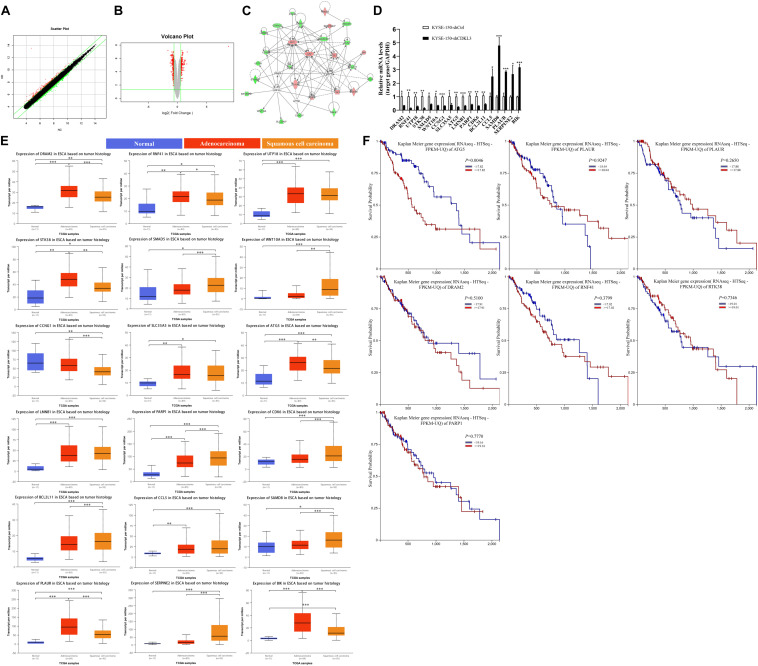
ATG5 is a potential target of CDKL3. **(A)** A scatter plot demonstrated the distribution of the signal intensity between two groups in a Cartesian coordinate plane. The *X*-axis represents KYSE-150-shCtrl group, and the *Y*-axis represents the KYSE-150-shCDKL3 group. The dots above the upper parallel line represented the downregulated genes, while the dots under the lower parallel line represented the upregulated genes. **(B)** Volcano Plot exhibited the significantly differentially expressed genes between the two groups. The *X*-axis denotes the log2-fold difference and the *Y*-axis denotes the log10-corrected significant. The red dots represented the differentially expressed genes screened with the absolute value of Fold Change ≥1.5 and FDR < 0.05. **(C)** The interaction network illustrated the interrelationships among these 22 selected genes surrounding the regulation of CDKL3. The up-regulation gene was red while the down-regulation gene was green. The solid line represented the direct interactions while the broken lines represented the indirect interactions, and an arrow represented the activation. **(D)** RT-PCR was used to confirm the up-regulated and down-regulated genes induced by CDKL3 knockdown in KYSE-150 cells. **(E)** The expression patterns of the potential interacting genes in the TCGA database according to tumor histology. **(F)** Comparison of overall survival of esophageal cancer patients with different genes expression based on TCGA data. Data were represented as mean ± SD. *P < 0.05, **P < 0.01, ***P < 0.001 vs. controls, respectively.

Scatter plot (Figure 5A) demonstrated the distribution of the signal intensity between KYSE-150-shCtrl and KYSE-150-shCDKL3 groups in the Cartesian coordinate plane. The downregulated genes were designated as the dots above the upper parallel line, while the upregulated genes were designated as the dots under the lower parallel line. Volcano plot (Figure 5B) was used to exhibit the significantly differentially expressed genes between the two groups. The red dots represented the differentially expressed genes screened with the absolute value of Fold Change ≥1.5 and FDR < 0.05. As a result, 22 genes (BCL2L11, RNF41, CCL5, DUSP6, UTP18, PLAUR, STK38, SLC35A5, CCNA2, ATG5, HMGA2, SMAD5, CTGF, SERPINE2, CCNG1, WNT10A, LMNB1, BIK, PARP1, CDK6, EIF4EBP2, and DDX58) were chosen for the illustration of the interrelationships among these molecules surrounding the regulation of CDKL3 by the Ingenuity Pathway Analysis (IPA) (Figure 5C). qRT-PCR was applied to verify these upregulated and downregulated genes selected by microarray analysis. As shown in Figure 5D, the mRNA levels of CCL5, SAMD8, PLAUR, SERPINE2, and BIK were significantly upregulated (2.500, 4.793, 2.862, 2.678, and 3.181 folds, respectively) in KYSE-150-shCDKL3 cells, whereas those of DRAM2, RNF41, UTP18, STK38, SMAD5, WNT10A, CCNG1, SLC35A5, ATG5, LMNB1, PARP1, CDK6, and BCL2L11 were significantly downregulated (0.355, 0.153, 0.284, 0.153, 0.45, 0.356, 0.326, 0.393, 0.249, 0.334, 0.395, 0.309, and 0.407 folds, respectively) in KYSE-150-shCDKL3 cells. The regulation of these genes was all consistent with results obtained from microarray analysis. However, the results of qRT-PCR showed no difference in expression of CTGF, HMGA2, and DDX58, and displayed a reverse trend of EIF4EBP2 expression. Then, UALCAN and TCGA datasets were employed to analyze the expression profile of these potential target genes of CDKL3 based on tumor histology. The statistically higher expressions of PLAUR, BIK, DRAM2, RNF41, STK38, ATG5, and PARP1 were measured in esophageal cancer than those in normal tissue, while statistically significant differences were also detected between ESCC and esophageal adenocarcinoma (EAC) (Figure 5E). Subsequently, UCSC Xena was applied to conduct survival analysis. The results showed that esophageal cancer patients with lower expression of ATG5 had a longer overall survival (Figure 5F). Autophagy-related gene 5 (ATG5) is identified as an essential autophagy gene, contributing to the malignant phenotype of cancer cells, and its higher expression is closely associated with poor prognosis in patients with early-stage ESCC (6–8). However, the association between ATG5 and CDKL3 in cancer has not been studied previously. Because of ATG5 upregulation as a potential marker of poor prognosis in ESCC, ATG5 was selected for further study in our project.

### Restoration of ATG5 Expression Counteracts the Effects of CDKL3

**FIGURE 6:**
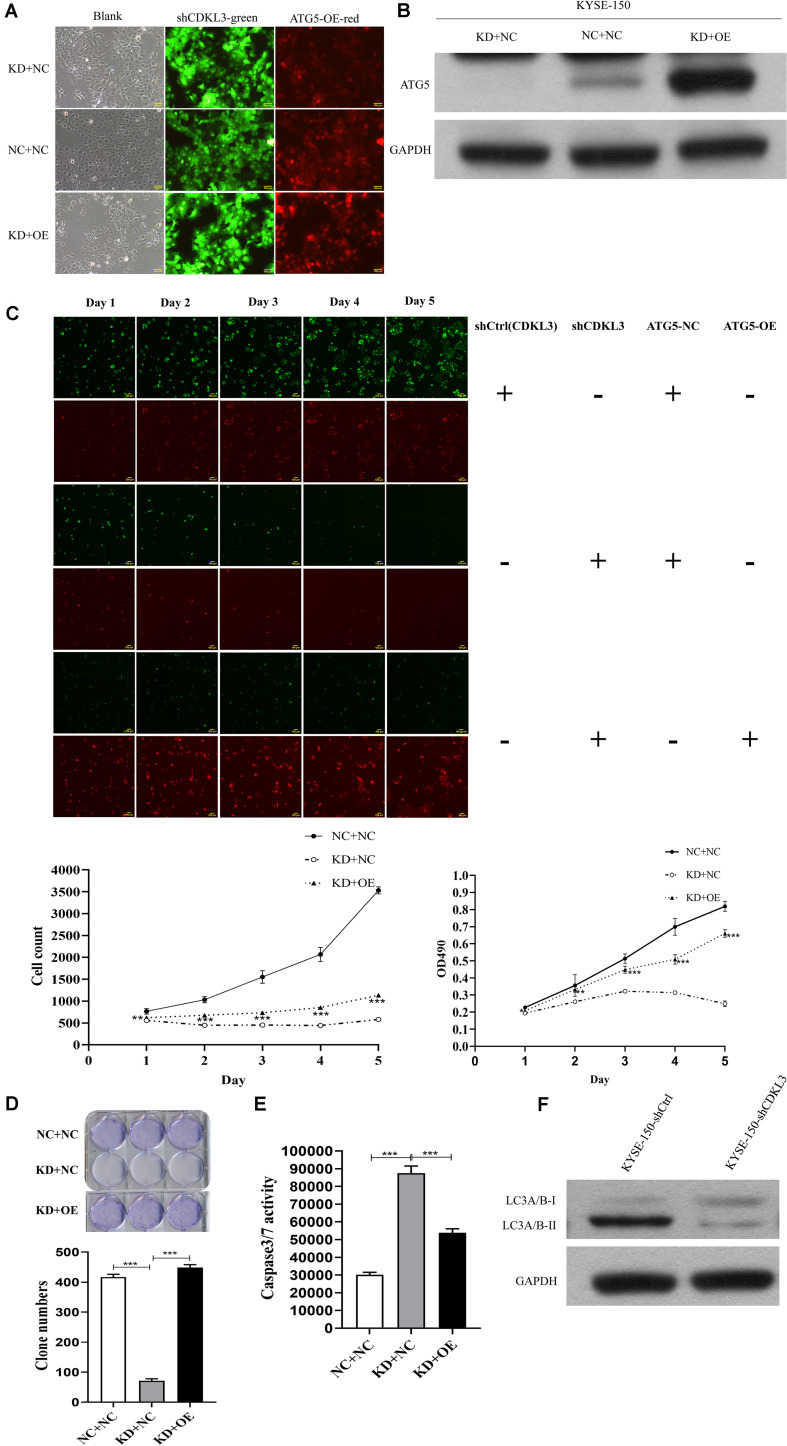
ATG5 re-expression partly counteracted the anti-tumor effect of CDKL3 knockdown in KYSE-150 cells. **(A)** After infection, KYSE-150 cells expressed green and red fluorescence, Magnification 100×. **(B)** Protein level showed that the re-overexpression of ATG5 in KD + OE group and down-expression of ATG5 in KD + NC-2 group (KD + NC). **(C)** Overexpressed ATG5 partially diminished the anti-tumor effect of CDKL3 knockdown by proliferation assays compared to KD + NC group. **(D)** The ability of colony formation was restored by ATG5 re-expression compared to KD + NC group. **(E)** ATG5 re-expression induced caspase-3/7 activity, which was inhibited by CDKL3 knockdown compared to KD + NC group. Data were represented as the mean ± SD of three separate experiments. *P < 0.05; **P < 0.01, ***P < 0.001. **(F)** The expression of LC3A/B was analyzed by western blot analysis.

To verify ATG5 is the functional target of CDKL3, rescue experiments were performed to investigate whether restoration of ATG5 expression counteracts the effects of CDKL3. After infection, the green and red fluorescence were visualized in over 80% of KYSE-150 cells (Figure 6A). Western blot analysis was conducted to uncover the re-expression of ATG5 in KD+OE group (Figure 6B). ATG5 re-expression weaken the tumor-inhibitive effect of CDKL3 knockdown in KD+OE group compared to KD+NC group (Figure 6C proliferation assays, *p* < 0.001, Figure 6D; colony formation, *P* < 0.001, Figure 6E caspase-3/7 activity assay, *p* < 0.001). Moreover, western blot analysis was performed to detect whether LC3A/B was regulated in response to CDKL3 knockdown. As shown in Figure 6F, the expression of LC3A/B, indicative of autophagy, was markedly attenuated in KYSE-150-shCDKL3 cells.

### The Higher-Expression of CDKL3 and ATG5 Is Associated With Poorer ESCC Prognosis

**FIGURE 7:**
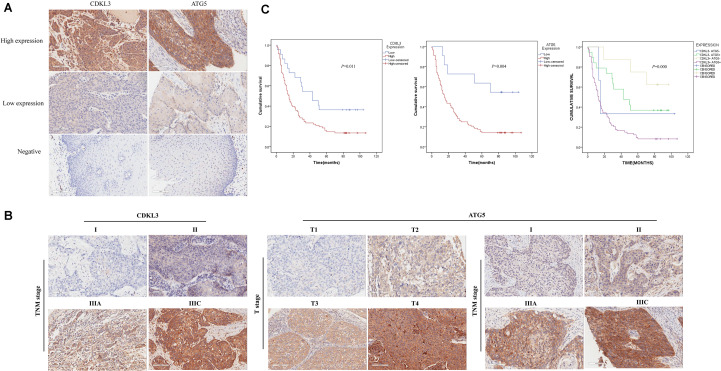
The expression of CDKL3 and ATG5 is upregulated in ESCC tissues and predicts a poor clinical outcome. **(A)** typical immunohistological pictures with high expressions of CDKL3 and ATG5 in ESCC. The staining showed predominantly cytoplasmic localization, Magnifications 200×. **(B)** Representative images of CDKL3 staining according to TNM stage and representative images of ATG5 staining according to the T stage and TNM stage in ESCC patients (magnification, 200×). **(C)** Kaplan–Meier survival curve was drawn to analyze the prognostic significance of CDKL3 and ATG5 expression in overall survival of ESCC patients.

**FIGURE 8:**
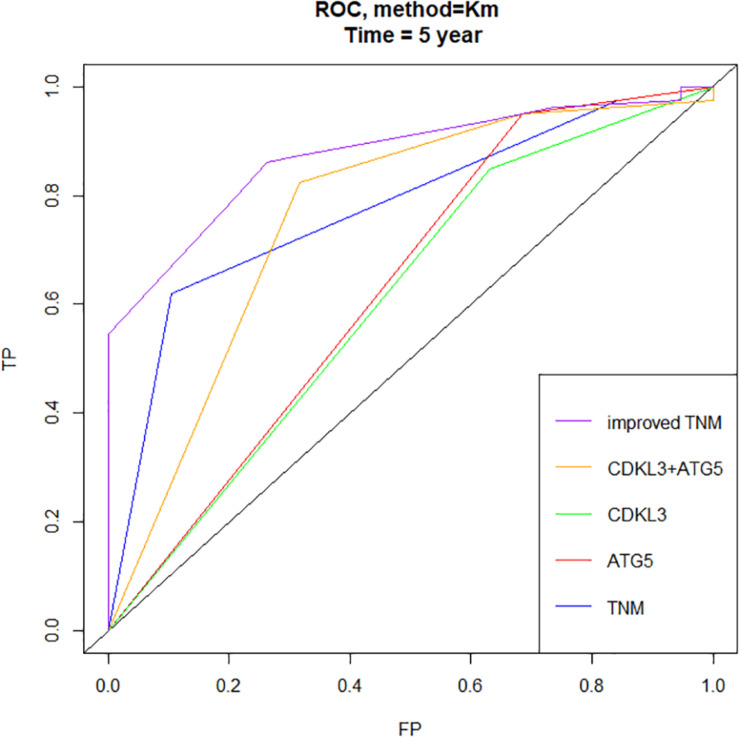
Time-dependent ROC curves showed that the combination of CDKL3, ATG5 and TNM stage–based model (named as improved TNM) had better predictive accuracy than TNM stage in ESCC patients (FP, false positive or specificity, TP, true positive or sensitivity).

The protein levels of CDKL3 and ATG5 in ESCC samples was examined by immunohistochemical analysis. Our results from 46 paired tumor and normal adjacent tissues indicated that CDKL3 and ATG5 were, respectively, high-expressed in 32 of 46 (69.56%) and 41 of 46 (89.13%) cases of ESCC, which was statistically higher than that in the adjacent non-tumor tissue (Figure 7A and Table 1). CDKL3 and ATG5 staining were both localized to the cytoplasm of the ESCC tissue cells. The clinicopathologic analysis (Table 2) revealed that high expressions of CDKL3 were mainly correlated with advanced TNM stage (*p* = 0.047) and Ki67 positive (*p* = 0.030). High ATG5 expression was correlated with gender (*p* = 0.000), T stage (*p* = 0.011) and TNM stage (*p* = 0.032). As shown in Figure 7B, the representative staining results of CDKL3 and ATG5 in ESCC patients with each T grade and TNM stage were statistically different the fluorescein staining results. Kaplan-Meier analysis indicated that ESCC patients with higher expression of CDKL3 and ATG5 had shorter overall survival (*p* = 0.011, *p* = 0.004, Figure 7C). Using Univariate Cox regression analysis, the results confirmed negative correlations between overall survival and gender (*p* = 0.014), tumor invasion (*p* = 0.000), lymph-node metastasis (*p* = 0.000), TNM stage (*p* = 0.000), and high CDKL3 (*p* = 0.014) and ATG5 expression (*p* = 0.007) in ESCC patients. Multivariate analysis by Cox’s proportional-hazard model further revealed that CDKL3 expression is an independent poor prognostic indicator in ESCC patients (HR 1.945; 95% CI 1.030–3.672; *p* = 0.040; Table 3). Basing on the expression of CDKL3 and ATG5 in ESCC tissues, 103 patients were further divided into 4 groups including group A (CDKL3-high and ATG5-high), group B (CDKL3-high and ATG5-low), group C (CDKL3-low and ATG5-high) and group D (CDKL3-low and ATG5-low). The ESCC patients with both high expressions of CDKL3 and ATG5 exhibited the worst prognosis for overall survival outcomes (*p* = 0.000, Figure 7C). Statistically, significant differences were investigated between some pairs of survival curves, containing group A and group B (*p* = 0.001); group B and group C (*p* = 0.001). Moreover, Spearman’s rank correlation coefficient displayed that there was no statistical association between the expression of CDKL3 and ATG5 in ESCC patients (Table 4). Besides, to investigate the predictive significance of CDKL3 and ATG5 in prognostic value of 5-year OS in ESCC patients, time-dependent ROC curve was analyzed to compare the predictive accuracy between the CDKL3-based nomogram, ATG5-based nomogram, CDKL3 + ATG5-based nomogram, CDKL3 + ATG5 + TNM stage–based nomogram (named as improved TNM) and TNM stage. As shown in Figure 8, the AUC values for CDKL3, ATG5, CDKL3 + ATG5, improved TNM and TNM stage were 0.608, 0.633, 0.76, 0.873, and 0.776, respectively. The better prognostic accuracy was found in improved TNM-based model than in TNM stage-based model (AUC: 0.873 vs. 0.776, *P* < 0.05). In summary, these results indicated that CDKL3 could be applied as an independent poor prognostic marker and might contribute to the malignant progression of ESCC. The combination of CDKL3 and ATG5 can be used as supplement to the current evaluation system of TNM stage to improve the predicting prognosis in ESCC patients.

**TABLE 1:** Differential expression of CDKL3 and ATG5 in 46 paired ESCC and adjacent tissues.

	CDKL3 expression		*p*	ATG5 expression		*p*
	High	Low		High	Low	
			0.034*			0.036*
Tumor tissues	32	14		41	5	
Adjacent tissues	22	24		33	13	

*P < 0.05.

**TABLE 2:** The relationship between CDKL3 and ATG5 expression status and clinicopathologic features of ESCC.

	clinicopathologic features	Total	CDKL3 expression		*p*	ATG5 expression		*p*
			Low	High		Low	High	
Age (years)					0.121			0.802
	≤65	50	14	36		5	45	
	>65	52	8	44		6	46	
Gender					0.423			0.000***
	Female	26	7	19		8	18	
	Male	77	15	62		3	74	
T stage					0.858			0.011*
	T1/T2	19	4	15		5	14	
	T3/T4	78	15	63		5	73	
N stage					0.125			0.180
	N0	46	13	33		7	39	
	N1/N2/N3	57	9	48		4	53	
TNM stage					0.047*			0.032*
	I/II	47	13	34		8	39	
	III/IV	51	6	45		2	49	
Differentiation grade					0.270			0.060
	1/2	80	19	61		11	69	
	3/4	23	3	20		0	23	
P53					0.549			0.876
	Negative	24	4	20		2	22	
	Positive	53	12	41		5	48	
Ki67					0.030*			0.825
	Negative	30	10	20		3	27	
	Positive	47	6	41		4	43	
PDL-1					0.959			0.724
	Negative	51	11	40		6	45	
	Positive	52	11	41		5	47	

T, tumor-metastasis; N, node-metastasis; TNM, tumor-node-metastasis; *P < 0.05; ***P < 0.001.

**TABLE 3:** Univariate and multivariate analyses of prognostic factors correlated with OS in ESCC patients.

	Clinicpathologic features	Overall survival		
		HR	95% CI	*p*
Univariate analysis	Gender (female vs. male)	1.995	1.152–3.453	0.014*
	Age (≤65 vs. >65)	1.004	0.980–1.028	0.740
	T stage (T1/T2 vs. T3/T4)	2.485	1.498–4.122	0.000***
	N stage (N0 vs. N1/N2/N3)	1.594	1.241–2.046	0.000***
	TNM stage (I/II vs. III/IV)	2.708	1.753–4.184	0.000***
	Differentiation grade (1/2 vs. 3/4)	0.825	0.543–1.252	0.365
	P53 (Negative vs. Positive)	0.743	0.434–1.270	0.277
	Ki67 (Negative vs. Positive)	1.165	0.695–1.951	0.562
	PDL-1 (Negative vs. Positive)	1.133	0.739–1.739	0.567
	CDKL3 expression (Low vs. High)	2.064	1.159–3.676	0.014*
	ATG5 expression (Low vs. High)	3.469	1.398–8.605	0.007**
Multivariate analysis	Gender (Female vs. male)	1.455	0.826–2.565	0.194
	T stage (T1/T2 vs. T3/T4)	1.994	1.033–3.847	0.040*
	N stage (N0 vs. N1/N2/N3)	1.234	0.831–1.833	0.298
	TNM stage (I/II vs. III/IV)	1.357	0.626–2.943	0.439
	CDKL3 expression (Low vs. High)	1.945	1.030–3.672	0.040*
Multivariate analysis	Gender (Female vs. male)	1.271	0.705–2.290	0.425
	T stage (T1/T2 vs. T3/T4)	1.756	0.891–3.461	0.104
	N stage (N0 vs. N1/N2/N3)	1.242	0.830–1.860	0.292
	TNM stage (I/II vs. III/IV)	1.537	0.705–3.354	0.280
	ATG5 expression (Low vs. High)	1.925	0.722–5.135	0.191

TNM, tumor-node-metastasis; HR, hazard ratio; CI, confidential interval; *P < 0.05; **P < 0.01; ***P < 0.001.

**TABLE 4:** Association of CDKL3 expression levels with ATG5 expression status.

		Total	CDKL3 expression		*p*	*r*
			Low	High		
ATG5 expression					0.916	0.011
	Low		3	8		
	High		19	73		

## Discussion

Our previous Vitro studies have revealed that CDKL3 plays an essential role in proliferation and metastasis of ESCC cell TE-1 and analysis of the TCGA dataset showed that CDKL3 function as poor prognosis in ESCC patients (3). However, the precise role, molecular mechanism, and realistic prediction of CDKL3 expression in ESCC have yet to be explored. CDKL3 works as an essential cell-cycle regulator similar to CDK3, and previous studies have revealed the regulation of CDKL3 in the enhancement of cellular proliferation in various engineering cell lines (9, 10). The abnormal expression of CDKL3 has been revealed to be related to human cancers (11–14). As far as we know, our study is the first to perform the profound and detailed investigation of CDKL3 in ESCC. Based on our previous research results, we conducted further examination to verify the role of CDKL3 *in vitro*, *in vivo*, and clinical prognosis in our ESCC patients with more clinical information in this article.

In this project, the results from Celigo and MTT analysis showed that ESCC cells with CDKL3 knockdown exhibit reduced proliferation capacity. CDKL3 knockdown enhanced apoptosis in ESCC cells by elevated caspase-3/7 activity. The ability of cellular colony formation, migration and invasion were also remarkably attenuated by CDKL3 knockdown in ESCC cells. The *in vivo* data showed the vital effect of CDKL3 on the tumorigenicity of ESCC. These data further affirmed the impact of CDKL3 on the malignant transformation and progression of ESCC. However, researches on the carcinogenesis modulation of CDKL3 rarely involved the molecular mechanisms in published studies. To address the potential molecular mechanisms of CDKL3 in carcinogenesis, we subsequently applied and analyzed microarray array to identify the differential gene expression profile regulated by CDKL3. After verification of qRT-PCR results and literature review, ATG5 was selected as a potential downstream effector of CDKL3 for further investigation. As we know, autophagy plays a vital role in various physiologic and pathologic processes containing tumorigenesis (15, 16). ATG5, as one of the critical regulators of autophagy, can be regulated by various genes contributing to tumorigenesis, tumor progression, and chemo-/radio-resistance (8, 17–20). However, the relation between ATG5 and CDKL3 in tumors was firstly revealed in our report. Re-expression of ATG5 in ESCC cells transfected with CDKL3-shRNA could partly reverse CDKL3-knockdown mediated repression of proliferation, apoptosis, and colony formation compared to ESCC cells treated with CDKL3-shRNA alone. Therefore, CDKL3 played a pivotal role in promoting tumor progression by ATG5 activation, which is a crucial gene involved in autophagy regulation. Although it has been proven that ATG5 can be used as a poor marker of poor prognosis in early-stage ESCC patients (6), the associations among ATG5, CDKL3, PDL-1, P53, and Ki67 status in different stages of ESCC patients remains unknown in the previous publication. Tumor tissues and adjacent normal tissues from ESCC patients were immunostained for expression of CDKL3 and ATG5, both of which were predominantly localized in the cytoplasm. Furthermore, the negative effect of CDKL3 and ATG5 on ESCC survival was highlighted by the Kaplan-Meier curve and Cox regression analysis. The data from multivariate Cox regression analysis suggested that CDKL3 could be used as independent prognostic factors in ESCC. The worst prognosis was reflected in patients with both high expressions of CDKL3 and ATG5. Unexpectedly, the best prognosis was not found in ESCC patients with CDKL3-low and ATG5-low, as a result of only three patients exhibiting both low expression of CDKL3 and ATG5 in tumor tissues. Another analysis was conducted to estimate the relation association among CDKL3, ATG5, PDL-1, Ki67, and p53. PD-L1 is expressed on the surface of tumor cells, and can aids in the evasion of antitumor immunity by binding to PD-1 (21, 22). Wang Q. et al. found that ESCC patients with PDL-1 over-expression who underwent surgical resection showed poor overall survival (23). In another report, the lack of PDL-1 expression was paradoxically associated with worse overall survival in ESCC patients who received esophagectomy (24). These contradictory results aroused our interest to analyze the association between PDL-1 and CDKL3. However, our data suggested that there was no statistically significant relation between PDL-1 and CDKL3 or ATG5. These discrepant results may be partially explained by the different cutoff values for PDL-1 positivity and the heterogeneity for PDL-1 expression in tumor cells. As compared with tumor cells, stromal cells such as tumor-infiltrating lymphocytes may be more appropriate for the analysis of PDL-1 expression. Wild type p53 considered as a key regulator in many cell-fate-determining processes and is mutated in nearly 50% of human tumors (25). A growing body of evidence suggested that mutation p53 was related to aggressiveness and poor prognosis (26). But no statistically significant association was found between p53 and CDKL3 or ATG5 in our study. The higher expression of Ki-67, well-known as a proliferation marker, heralds worse tissue differentiation, and poorer prognosis in many human tumors (27). Our analysis revealed no statistical association between Ki67 and ATG5, whereas a statistically significant higher expression of CDKL3 was related to Ki67 positive ESCC patients. The relation between CDKL3 and Ki67 has not been explored in previous studies. To develop a prognostic model of CDKL3 and Ki67 may be one of the further research directions in our advance project.

## Conclusion

Collectively, CDKL3 has been identified as an independent poor prognostic marker in ESCC patients, and ESCC patients with both high expressions of CDKL3 and ATG5 had the worst survival. Mechanistic detection demonstrated that CDKL3 might promote tumor profession, invasion, metastasis, and prohibit tumor apoptosis partly by ATG5 activation. These findings firstly depict that the elevated ATG5 expression is a significant gene involving in the tumor-promoting effect of CDKL3 in ESCC, suggesting that ATG5 and CDKL3 may be applied as a therapeutic target for ESCC.

## Data Availability Statement

The datasets presented in this study can be found in online repositories. The names of the repository/repositories and accession number(s) can be found below: the NCBI Gene Expression Omnibus (GSE154945).

## Ethics Statement

The studies involving animals were reviewed and approved by the Institutional Animal Care and Use Committee (IACUC) of Wenzhou Medical University.

## Author Contributions

HY and WY conceived and participated in the design of the study. SZ and MZ wrote and revised the manuscript. CZ recruited samples. SZ, CZ, and WW performed all the experimental work. SZ, MZ, WY, and HY participated in data analysis. All authors read and approved the final manuscript.

## Conflict of Interest

The authors declare that the research was conducted in the absence of any commercial or financial relationships that could be construed as a potential conflict of interest.

## Supplementary Material

FIGURE S1 The valuation of the quality of the microarray data. **(A)** The signal intensity distribution graph showed the signal intensity distribution of all the chip probes. The better the coincidence of the signal intensity distribution curves of different samples, the higher the reliability of the chip results. **(B)** Box plot of relative log signal strength illustrated the distribution of normalized log-transformed expression. The closer the distribution, the better the repeatability of the data. **(C)** Pearson correlation coefficient distribution chart displayed the correlation level of signal strength among all chips [−1.0,1.0]. The red clump represented as higher correlation; the blue clump represented as lower correlation in the distribution diagram of the correlation coefficient. **(D)** Principal Component Analysis (PCA) was a quasi-statistical technique for forming a new set of linearly transformed variables. In the PC1 and PC2 dimensions, the red dots represented as shCDKL3 (KD) samples and green dots represented as shCtrl (NC) samples.
