# Corrigendum: CDKL3 Targets ATG5 to Promote Carcinogenesis of Esophageal Squamous Cell Carcinoma

**DOI:** 10.3389/fonc.2020.619438

**Published:** 2021-03-04

**Authors:** Suna Zhou, Mingxin Zhang, Chao Zhou, Wei Wang, Haihua Yang, Wenguang Ye

**Affiliations:** ^1^ Laboratory of Cellular and Molecular Radiation Oncology, The Affiliated Taizhou Hospital, Wenzhou Medical University, Taizhou, China; ^2^ Department of Radiation Oncology, The Affiliated Taizhou Hospital, Wenzhou Medical University, Taizhou, China; ^3^ Department of Gastroenterology, The First Affiliated Hospital of Xi’an Medical University, Xi’an, China; ^4^ Department of Gastroenterology, The Affiliated Taizhou Hospital, Wenzhou Medical University, Taizhou, China

**Keywords:** cyclin-dependent kinase-like 3, autophagy-related gene 5, esophageal squamous cell carcinoma, prognosis, carcinogenesis

In the original article, there was an error in the **Methods** section of the **Abstract**. We carelessly wrote FLVCR1 instead of CDKL3.

A correction has been made to the **Methods** section of the **Abstract**, paragraph 2:

“**Methods**: ESCC samples were stained by immunohistochemical staining (IHC) and analyzed for the expression of CDKL3. The functions of CDKL3 on proliferation, apoptosis, migration, invasion, and colony formation were investigated by celigo assay, MTT assay, colony formation, caspase 3/7 activity analysis, transwell migration and invasion assay, respectively. A transplanted tumor model was established to study the functions of CDKL3 on the tumorigenesis of ESCC cells. Microarray analysis was utilized to identify the CDKL3-regulated genes in ESCC cells.”

In the original article, there was a mistake in the legend for FIGURE 5(F) as published. “genes” was erroneously written as “CSE1L/RPS15A/SFPQ/CAPZB”. The correct legend appears below.

“**Figure 5.** ATG5 is a potential target of CDKL3. (A) A scatter plot demonstrated the distribution of the signal intensity between two groups in a Cartesian coordinate plane. The X-axis represents KYSE-150-shCtrl group, and the Y-axis represents the KYSE-150-shCDKL3 group. The dots above the upper parallel line represented the downregulated genes, while the dots under the lower parallel line represented the upregulated genes. (B) Volcano Plot exhibited the significantly differentially expressed genes between the two groups. The X-axis denotes the log2-fold difference and the Y-axis denotes the log10-corrected significant. The red dots represented the differentially expressed genes screened with the absolute value of Fold Change ≥1.5 and FDR < 0.05. (C) The interaction network illustrated the interrelationships among these 22 selected genes surrounding the regulation of CDKL3. The up-regulation gene was red while the down-regulation gene was green. The solid line represented the direct interactions while the broken lines represented the indirect interactions, and an arrow represented the activation. (D) RT-PCR was used to confirm the up-regulated and down-regulated genes induced by CDKL3 knockdown in KYSE-150 cells. (E) The expression patterns of the potential interacting genes in the TCGA database according to tumor histology. (F) Comparison of overall survival of esophageal cancer patients with different genes expression based on TCGA data. Data were represented as mean ± SD. *P < 0.05, **P < 0.01, ***P < 0.001 vs. controls, respectively.”

The authors apologize for these errors and state that this does not change the scientific conclusions of the article in any way. The original article has been updated.

