# Supplementary figures and images for: CDKL3 Targets ATG5 to Promote Carcinogenesis of Esophageal Squamous Cell Carcinoma
Source: Front Oncol. 2020 Aug 21;10:1602. doi: 10.3389/fonc.2020.01602 (PMC7473399; doi:10.3389/fonc.2020.01602)

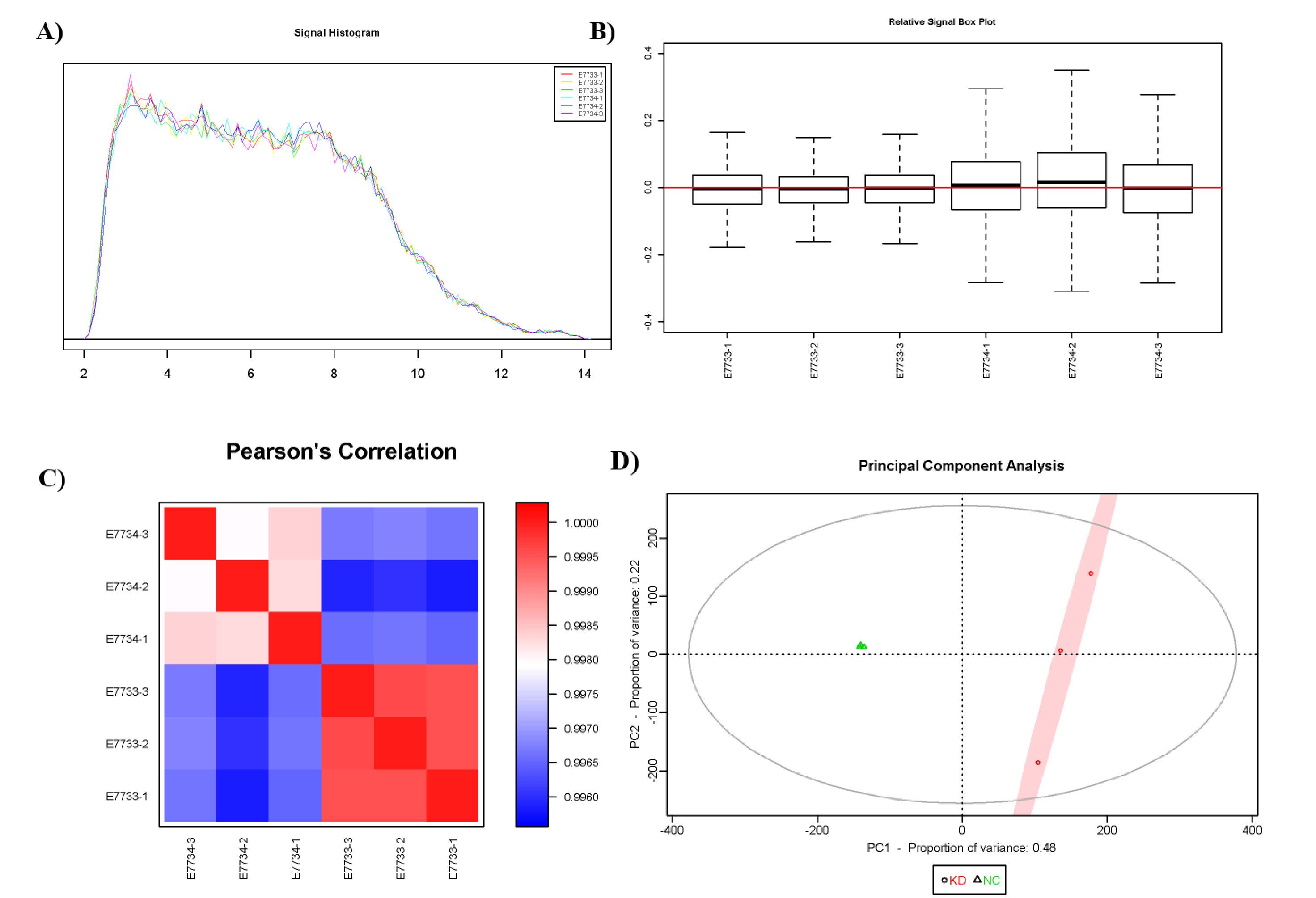

Supplement: FIGURE S1 — The valuation of the quality of the microarray data. (A) The signal intensity distribution graph showed the signal intensity distribution of all the chip probes. The better the coincidence of the signal intensity distribution curves of different samples, the higher the reliability of the chip results. (B) Box plot of relative log signal strength illustrated the distribution of normalized log-transformed expression. The closer the distribution, the better the repeatability of the data. (C) Pearson correlation coefficient distribution chart displayed the correlation level of signal strength among all chips [−1.0,1.0]. The red clump represented as higher correlation; the blue clump represented as lower correlation in the distribution diagram of the correlation coefficient. (D) Principal Component Analysis (PCA) was a quasi-statistical technique for forming a new set of linearly transformed variables. In the PC1 and PC2 dimensions, the red dots represented as shCDKL3 (KD) samples and green dots represented as shCtrl (NC) samples. [file Image_1.tif]
